# Supplementary material for: DNA microarray analysis of Staphylococcus aureus from Nigeria and South Africa
Source: PLoS One. 2021 Jul 20;16(7):e0237124. doi: 10.1371/journal.pone.0237124 (PMC8291685; doi:10.1371/journal.pone.0237124)
Supplement: S4 Table — (DOC) [file pone.0237124.s004.doc]

**Distribution of selected genes (DNA microarray) in *S. aureus* isolates from Nigeria and South Africa.**

| **CATEGORY** | **GENE** | **GENE PRODUCT/FUNCTION** | **t037**  **CC8 (n=8)** | **t045**  **CC5**  **(n=5)** | **t064**  **CC8**  **(n=17)** | **t951**  **CC8**  **(n=1)** | **t1257**  **CC8**  **(n=6)** | **t2723**  **CC88**  **(n=1)** | **t6238**  **CC8**  **(n=1)** | **NT**  **CC8**  **(n=1)** |
| --- | --- | --- | --- | --- | --- | --- | --- | --- | --- | --- |
| **RESISTANCE: PENICILLINASE** | *blaZ* | Beta-lactamase |  |  |  |  |  |  |  |  |
|  | *blaI* | Beta-lactamase repressor (inhibitor) |  |  |  |  |  |  |  |  |
|  | *blaR* | Beta-lactamase regulatory protein |  |  |  |  |  |  |  |  |
| **METHICILLIN RESISTANCE** | *mecA* | Alternate penicillin-binding protein 2, defining MRSA |  |  |  |  |  |  |  |  |
| **RESISTANCE: MACROLIDE-LINCOSAMIDE-STREPTOGRAMIN B ANTIBIOTICS** | *ermA* | rRNA adenine N-6-methyltransferase |  |  |  |  |  |  |  |  |
|  | *ermB* | Erythromycin/clindamycin resistance |  |  |  |  |  |  |  |  |
|  | *ermC* | Erythromycin/clindamycin resistance |  |  |  |  |  |  |  |  |
|  | *lin(A)* | Lincosamide nucleotidyltransferase A |  |  |  |  |  |  |  |  |
|  | *msr(A)* | Energy-dependent efflux of erythromycin |  |  |  |  |  |  |  |  |
|  | *mef(A)* | Macrolide efflux protein A |  |  |  |  |  |  |  |  |
|  | *vat(A)* | Virginiamycin A acetyltransferase |  |  |  |  |  |  |  |  |
|  | *vat(B)* | Acetyltransferase inactivating streptogramin A |  |  |  |  |  |  |  |  |
|  | *vga(A)* | ATP binding protein, streptogramin A resistance |  |  |  |  |  |  |  |  |
| **RESISTANCE: AMINOGLYCOSIDES** | *aacA-aphD* | Bi-functional aminoglycoside adenyl-/phosphotransferase |  |  |  |  |  |  |  |  |
|  | *aadD* | Aminoglycoside adenyltransferase |  |  |  |  |  |  |  |  |
|  | *aphA3* | 3’-5’ aminoglycoside phosphotransferase |  |  |  |  |  |  |  |  |
| **RESISTANCE: MISCELLANEOUS GENES** | *sat* | Streptothricin acetyltransferase |  |  |  |  |  |  |  |  |
|  | *dfrA* | Dihydrofolate reductase |  |  |  |  |  |  |  |  |
|  | *far1* | Fusidic acid resistance gene |  |  |  |  |  |  |  |  |
|  | *Q6GD50 (fusC)* | Hypothetical protein associated with fusidic acid resistance |  |  |  |  |  |  |  |  |
|  | *mupR* | Mupirocin resistance protein |  |  |  |  |  |  |  |  |
|  | *tet(K)* | Tetracycline resistance gene |  |  |  |  |  |  |  |  |
|  | *tet(M)* | Tetracycline resistance gene |  |  |  |  |  |  |  |  |
|  | *cat* | Chloramphenicol acetyltransferase |  |  |  |  |  |  |  |  |
|  | *cfr* | 23S rRNA methyltransferase |  |  |  |  |  |  |  |  |
|  | *fexA* | Chloramphenicol/florfenicol exporter gene |  |  |  |  |  |  |  |  |
|  | *fosB* | Metallothiol transferase |  |  |  |  |  |  |  |  |
| **RESISTANCE: EFFLUX SYSTEMS** | *qacA* | Quaternary ammonium compound/multidrug efflux protein A |  |  |  |  |  |  |  |  |
|  | *qacC* | Quaternary ammonium compound/multidrug efflux protein C |  |  |  |  |  |  |  |  |
|  | *tetEfflux* | Transport/efflux protein |  |  |  |  |  |  |  |  |
|  | *mer* | Mercury resistance gene operon |  |  |  |  |  |  |  |  |
| **RESISTANCE: GLYCOPEPETIDES** | *vanA* | Vancomycin resistance gene |  |  |  |  |  |  |  |  |
|  | *vanB* | Vancomycin resistance gene from enterococci and Clostridium |  |  |  |  |  |  |  |  |
|  | *vanZ* | Teicoplanin resistance gene from enterococci |  |  |  |  |  |  |  |  |
| **VIRULENCE: TOXIC SHOCK TOXIN** | *tst1* | Toxic shock syndrome toxin |  |  |  |  |  |  |  |  |
| **VIRULENCE: ENTEROTOXINS** | *entA* | Enterotoxin A |  |  |  |  |  |  |  |  |
|  | *entB* | Enterotoxin B |  |  |  |  |  |  |  |  |
|  | *entC* | Enterotoxin C |  |  |  |  |  |  |  |  |
|  | *entD* | Enterotoxin D |  |  |  |  |  |  |  |  |
|  | *entE* | Enterotoxin E |  |  |  |  |  |  |  |  |
|  | *entG* | Enterotoxin G |  |  |  |  |  |  |  |  |
|  | *entH* | Enterotoxin H |  |  |  |  |  |  |  |  |
|  | *entI* | Enterotoxin I |  |  |  |  |  |  |  |  |
|  | *entJ* | Enterotoxin J |  |  |  |  |  |  |  |  |
|  | *entK* | Enterotoxin K |  |  |  |  |  |  |  |  |
|  | *entL* | Enterotoxin L |  |  |  |  |  |  |  |  |
|  | *entM* | Enterotoxin M |  |  |  |  |  |  |  |  |
|  | *entN* | Enterotoxin N |  |  |  |  |  |  |  |  |
|  | *entO* | Enterotoxin O |  |  |  |  |  |  |  |  |
|  | *egc* | egc cluster |  |  |  |  |  |  |  |  |
|  | *entQ* | Enterotoxin Q |  |  |  |  |  |  |  |  |
|  | *entR* | Enterotoxin R |  |  |  |  |  |  |  |  |
|  | *entU* | Enterotoxin U and/or Y |  |  |  |  |  |  |  |  |
| **VIRULENCE: LEUKOCIDINS AND**  **HAEMOLYSINS** | *lukF* | Haemolysin gamma/leukocidin, component B (F) |  |  |  |  |  |  |  |  |
|  | *lukS* | Haemolysin gamma/leukocidin, component C (S) |  |  |  |  |  |  |  |  |
|  | *hlgA* | Haemolysin gamma, component A |  |  |  |  |  |  |  |  |
|  | *lukF/S-PV* | Panton-Valentine Leukocidin (F/S component) |  |  |  |  |  |  |  |  |
|  | *lukF-PV* (*P83*) | F component of leukocidin from ruminants |  |  |  |  |  |  |  |  |
|  | *lukM* | Leukocidin S component |  |  |  |  |  |  |  |  |
|  | *lukD* | Leukocidin D component |  |  |  |  |  |  |  |  |
|  | *lukE* | Leukocidin E component |  |  |  |  |  |  |  |  |
|  | *lukX* | Leukocidin/haemolysin toxin family protein |  |  |  |  |  |  |  |  |
|  | *lukY* | Leukocidin/haemolysin toxin family protein |  |  |  |  |  |  |  |  |
|  | *hla* | Haemolysin alpha |  |  |  |  |  |  |  |  |
|  | *hld* | Haemolysin delta |  |  |  |  |  |  |  |  |
| **VIRULENCE: GENES ASSOCIATED WITH HEMOLYSIN BETA CONVERTING PHAGES** | *sak* | Staphylokinase |  |  |  |  |  |  |  |  |
|  | *chp* | Chemotaxis-inhibiting protein (CHIPS) |  |  |  |  |  |  |  |  |
|  | *scn* | Staphylococcal complement inhibitor |  |  |  |  |  |  |  |  |
| **VIRULENCE: EXFOLIATIVE TOXINS** | *etA* | Exfoliative toxin A |  |  |  |  |  |  |  |  |
|  | *etB* | Exfoliative toxin B |  |  |  |  |  |  |  |  |
|  | *etD* | Exfoliative toxin D |  |  |  |  |  |  |  |  |
| **VIRULENCE: EPITHELIAL DIFFERENTIATION INHIBITOR & ACME** | *edinA* | Epidermal cell differentiation inhibitor |  |  |  |  |  |  |  |  |
|  | *edinB* | Epidermal cell differentiation inhibitor B |  |  |  |  |  |  |  |  |
|  | *edinC* | Epidermal cell differentiation inhibitor C |  |  |  |  |  |  |  |  |
|  | ACME | Arginine catabolic mobile element |  |  |  |  |  |  |  |  |
| **VIRULENCE: PROTEASES** | *aur* | Aureolysin |  |  |  |  |  |  |  |  |
|  | *aur OtherThan252* | Aureolysin from other strains (not MRSA252) |  |  |  |  |  |  |  |  |
|  | *aur-MRSA252* | Aureolysin from strain MRSA252 |  |  |  |  |  |  |  |  |
|  | *splA* | Serine protease A |  |  |  |  |  |  |  |  |
|  | *splB* | Serine protease B |  |  |  |  |  |  |  |  |
|  | *splE* | Serine protease E |  |  |  |  |  |  |  |  |
|  | *sspA* | Glutamyl endopeptidase |  |  |  |  |  |  |  |  |
|  | *sspB* | Staphopain B, protease |  |  |  |  |  |  |  |  |
|  | *sspP* | Staphopain A (staphopain A) protease |  |  |  |  |  |  |  |  |
| **CAPSULE- AND BIOFILM-ASSOCIATED GENES** | *cap1* | Capsule type 1 |  |  |  |  |  |  |  |  |
|  | *cap5* | Capsule type 5 |  |  |  |  |  |  |  |  |
|  | *cap8* | Capsule type 8 |  |  |  |  |  |  |  |  |
|  | *icaA* | Intercellular adhesion A |  |  |  |  |  |  |  |  |
|  | *icaC* | Intercellular adhesion C |  |  |  |  |  |  |  |  |
|  | *icaD* | Intercellular adhesion D |  |  |  |  |  |  |  |  |
|  | *bap* | Surface protein involved in biofilm formation |  |  |  |  |  |  |  |  |

| **CATEGORY** | **GENE** | **GENE PRODUCT/FUNCTION** | **t037**  **CC8 (n=8)** | **t045**  **CC5**  **(n=5)** | **t064**  **CC8**  **(n=17)** | **t951**  **CC8**  **(n=1)** | **t1257**  **CC8**  **(n=6)** | **t2723**  **CC88**  **(n=1)** | **t6238**  **CC8**  **(n=1)** | **NT**  **CC8**  **(n=1)** |
| --- | --- | --- | --- | --- | --- | --- | --- | --- | --- | --- |
| **MICROBIAL SURFACE COMPONENTS RECOGNIZING ADHESIVE MATRIX MOLECULES (MSCRAMM) GENES** | *bbp* | Bone sialoprotein-binding protein |  |  |  |  |  |  |  |  |
|  | *clfA* | clumping factor A |  |  |  |  |  |  |  |  |
|  | *clfB* | Clumping factor B |  |  |  |  |  |  |  |  |
|  | *cna* | Collagen-binding adhesion |  |  |  |  |  |  |  |  |
|  | *ebpS* | Cell surface elastin binding protein |  |  |  |  |  |  |  |  |
|  | *eno* | Enolase |  |  |  |  |  |  |  |  |
|  | *fib* | Fibrinogen-binding protein A (19kDa) |  |  |  |  |  |  |  |  |
|  | *fnbA* | Fibronectin-binding protein A |  |  |  |  |  |  |  |  |
|  | *fnbB* | Fibronectin-binding protein B |  |  |  |  |  |  |  |  |
|  | *map* | Major histocompatibility complex II analogue protein |  |  |  |  |  |  |  |  |
|  | *sasG* | Staphylococcus aureus surface protein G |  |  |  |  |  |  |  |  |
|  | *sdrC* | Ser-Asp rich fibrinogen/bone sialoprotein-binding protein C |  |  |  |  |  |  |  |  |
|  | *sdrD* | Ser-Asp rich fibrinogen/bone sialoprotein-binding protein D |  |  |  |  |  |  |  |  |
|  | *vwb* | van Willebrand factor binding protein |  |  |  |  |  |  |  |  |
| **ACCESSORY GENE REGULATOR ALLELES (AGR GROUP AFFILIATION)** | *agr*I | Accessory gene regulatory allele I |  |  |  |  |  |  |  |  |
|  | *agr*II | Accessory gene regulatory allele II |  |  |  |  |  |  |  |  |
|  | *agr*III | Accessory gene regulatory allele III |  |  |  |  |  |  |  |  |
|  | *agr*IV | Accessory gene regulatory allele IV |  |  |  |  |  |  |  |  |

0% ≤20% 40-50% 60-70% 70-80% 80-90% 90-99% 100%
