# Supplementary material for: DNA microarray analysis of Staphylococcus aureus from Nigeria and South Africa
Source: PLoS One. 2021 Jul 20;16(7):e0237124. doi: 10.1371/journal.pone.0237124 (PMC8291685; doi:10.1371/journal.pone.0237124)
Supplement: Attachment — Submitted filename: Response to Reviewers_R3.doc [file pone.0237124.s005.doc]

Institute of Medical Microbiology

University Hospital Münster

Domagkstraße 10, 48149

Münster, Germany

23/04/2021

Professor Monica Cartelle Gestal

Academic Editor

PLoS One

Dear Professor Gestal,

**Re: Revised manuscript: PONE-D-20-22325-R3 – DNA microarray analysis of *Staphylococcus aureus* from Nigeria and South Africa**

On behalf of other co-authors, I forward herewith the revised manuscript for your kind consideration. The Journal requirements and comments of reviewer 2 have been examined and we provide a point-by-point response (in italics) as indicated below.

**Journal Requirements**

Please review your reference list to ensure that it is complete and correct. If you have cited papers that have been retracted, please include the rationale for doing so in the manuscript text, or remove these references and replace them with relevant current references. Any changes to the reference list should be mentioned in the rebuttal letter that accompanies your revised manuscript. If you need to cite a retracted article, indicate the article’s retracted status in the References list and also include a citation and full reference for the retraction notice.

*Response: Based on comments/suggestions from the reviewers, the under-listed references were included in the revised manuscript which was not in the original version.*

*9. Franklin R. Cockerill, III M, Jean B. Patel, PhD D. M100-S25 Performance Standards for Antimicrobial Susceptibility Testing; Twenty-Fifth Informational Supplement. Clin Lab Stand Inst. 2015.*

*10. Al-Haqan A, Boswihi SS, Pathan S, Udo EE. Antimicrobial resistance and virulence determinants in coagulase-negative staphylococci isolated mainly from preterm neonates. PLoS One. 2020. doi:10.1371/journal.pone.0236713.*

*13. Monecke S, Coombs G, Shore AC, Coleman DC, Akpaka P, Borg M, et al. A field guide to pandemic, epidemic and sporadic clones of methicillin-resistant Staphylococcus aureus. PLoS One. 2011. doi:10.1371/journal.pone.0017936.*

*14. Monecke S, Jatzwauk L, Müller E, Nitschke H, Pfohl K, Slickers P, et al. Diversity of SCCmec elements in staphylococcus aureus as observed in south-eastern Germany. PLoS One. 2016. doi:10.1371/journal.pone.0162654.*

*15. Landis JR, Koch GG. The Measurement of Observer Agreement for Categorical Data. Biometrics. 1977. doi:10.2307/2529310.*

*The reference number 10 in the original manuscript: Monecke S, Jatzwauk L, Weber S, Slickers P, Ehricht R. DNA microarray-based genotyping of methicillin-resistant Staphylococcus aureus strains from Eastern Saxony. Clinical Microbiology and Infection. 2008. doi:10.1111/j.1469-0691.2008.01986.x was replaced with the reference below.*

*12. Monecke S, Slickers P, Ehricht R. Assignment of Staphylococcus aureus isolates to clonal complexes based on microarray analysis and pattern recognition. FEMS Immunol Med Microbiol. 2008. doi:10.1111/j.1574-695X.2008.00426.x.*

*The reference list is complete and correct in the revised manuscript.*

**Reviewer’s comments**

Reviewer #2: This is the third review for the manuscript by Shittu et al., which describes the screening of 40 S. aureus isolates collected in South Africa from 2001-2004 for a variety of virulence factors and antimicrobial elements using DNA microarray analysis. This paper basically summarizes the findings of the screen and the concludes that “DNA microarray assay provides information on antibiotic resistance and virulence gene determinants and can be a useful tool to identify gene markers of specific S. aureus clones in Africa”. While the authors have made revisions to accommodate suggestions posed by reviewers, the authors fail to accommodate the request to provide a list of all target or gene names, along with the actual DNA sequence of the probes and primers. Therefore, the manuscript in its current form fails to meet PLoS One Data availability requirements. Instead of simply complying with the policy, the authors choose to argue that 1) previous published papers have not met the policy requirements and 2) that references listed provide the requested information.

This reviewer’s response to these points are:
1) This reviewer was not part of the peer-review process of the noncompliant previous published papers. In contrast, this reviewer is part of the peer-review process for the manuscript by Shittu et al., which in its current form, is NOT compliant.
2) The authors are correct in that one of the cited references provides the required information however, it is provided in a convoluted style. For example, the gene aacA-aphD, a bifunctional enzyme Aac/Aph conferring gentamicin resistance, the probe name is “hp_aacA-59243_PM1” and the probe sequence is “AB049452.1[5151:5178]”

This reviewer is unsure why the authors persist to refuse to provide a list of all target or gene names, along with the actual DNA sequence of the probes and primers, which would fulfill PLoS One Data availability requirements. These requirements are not meant to be a cumbersome obstacle. They are meant to help make all aspects of the scientific process accessible to everyone.

***Response****: We have included the primer and probe names, GenBank coordinates and the corresponding DNA sequences in the revised manuscript (Supplementary data: S1 Table 3). The DNA sequences for the primers and probes were retrieved from the NCBI website* [*https://www.ncbi.nlm.nih.gov/nuccore*](https://www.ncbi.nlm.nih.gov/nuccore) *through their accession numbers and coordinates, as published previously.*

*Monecke S, Coombs G, Shore AC, Coleman DC, Akpaka P, Borg M, et al. A field guide to pandemic, epidemic and sporadic clones of methicillin-resistant Staphylococcus aureus. PLoS One. 2011.* *doi: 10.1371/journal.pone.0017936.*

*Monecke S, Jatzwauk L, Müller E, Nitschke H, Pfohl K, Slickers P, et al. Diversity of SCCmec elements in Staphylococcus aureus as observed in south-eastern Germany. PLoS One. 2016.* *doi: 10.1371/journal.pone.0162654.*

Yours sincerely,

Professor Adebayo Shittu

Corresponding author
